# Efficacy of Kangfuxin liquid for preventing and treating chemotherapy-induced oral mucositis: a systematic review and meta-analysis of randomized controlled trials

**DOI:** 10.3389/fphar.2025.1565345

**Published:** 2025-04-08

**Authors:** Wei Sun, Yang Jian, Xiaolin Feng, Minru Zhao, Yuan Liu

**Affiliations:** ^1^ Department of Laboratory Medicine, The General Hospital of Western Theater Command, Chengdu, China; ^2^ Department of Clinical Pharmacy, The General Hospital of Western Theater Command, Chengdu, China

**Keywords:** Kangfuxin Liquid, oral mucositis, chemotherapy, systematic review, meta-analysis

## Abstract

**Objective:**

Chemotherapy-induced oral mucositis (CTOM) is a common side effect affecting 20%–40% of cancer patients receiving chemotherapy. Kangfuxin liquid (KFXL) has been used clinically to prevent and treat CTOM, but the evidence has not been systematically evaluated. This study aimed to evaluate the preventive and therapeutic effects of KFXL on CTOM.

**Methods:**

Nine electronic databases were searched to identify KFXL-related randomized controlled trials (RCTs) for the prevention and treatment of CTOM from inception to September 2024. The primary outcomes were incidence rate, efficacy rate and cure rate, and the secondary outcomes was healing time.

**Results:**

Twenty-one trials involving 1825 patients were included in this review. The results of our meta-analysis showed that, compared with basic oral care (BOC), KFXL significantly reduced the incidence rate of CTOM and severe CTOM (RR = 0.54, p < 0.00001; RR = 0.23, p < 0.00001, respectively), improved the efficacy rate of CTOM and severe CTOM (RR = 1.23, p = 0.0003; RR = 1.99, p = 0.05, respectively), improved the cure rate of CTOM (RR = 2.06, p = 0.0004),and accelerated the healing process (MD = −2.48, p < 0.00001). However, KFXL and other drugs have the same efficacy rate in treating CTOM and severe CTOM (RR = 1.00, p = 0.99; RR = 1.00, p = 1.00, respectively), and the same cure rate in CTOM (RR = 0.91, p = 0.39), and the same healing time (MD = −0.01, p = 1.00).

**Conclusion:**

The results suggest that KFXL may provide more benefit in the prevention and treatment for CTOM compared to BOC. Although KFXL may be a promising drug for the prevention and treatment of CTOM, the evidence is insufficient to prove its superiority over other guideline-recommended treatment.

**Systematic Review Registration:**

https://www.crd.york.ac.uk/PROSPERO/view/CRD42024585859, ID: CRD42024585859.

## 1 Introduction

Cancer remains the leading cause of death worldwide, and the side effects of its treatment seriously affect patients’ quality of life and prognosis, as well as increasing treatment costs (Lee et al., 2024). “Mucositis” is a MeSH term that describes inflammation of the mucosa as a result of chemotherapy and/or radiotherapy. Chemotherapy-induced oral mucositis (CTOM) presents as erythema and ulceration of the oral mucosa and is pathologically characterized by vascular damage, atrophy of the squamous epithelium, inflammatory infiltration, and ulceration (Peterson et al., 2015). CTOM is a painful condition that can significantly affect food intake, oral hygiene, communication and quality of life (Lalla et al., 2008). Severe oral mucositis can lead to dose reductions, delays and/or discontinuation of cancer treatment, resulting in a poor prognosis (Jensen and Peterson, 2014). The incidence of CTOM was approximately 20%–40% in patients receiving conventional chemotherapy and 80% in patients receiving high-dose chemotherapy as a prerequisite for hematopoietic stem cell transplantation (Jones et al., 2006; Vera-Llonch et al., 2007). A number of grading scales have been developed to characterize the severity of CTOM. Two of the most commonly used scales for CTOM are the WHO (World Health Organization) and NCI-CTCAE (The National Cancer Institute Common Terminology Criteria for Adverse Events scales) (Peterson et al., 2015). The main goals of these managements are to reduce the incidence, intensity and duration of CTOM, and to provide symptomatic relief (Villa and Sonis, 2016). The Multinational Association of Supportive Care in Cancer and International Society of Oral Oncology (MASCC/ISOO) has published detailed clinical practice guidelines for the management of OM: BOC, anti-inflammatory, laser and other light therapy; cryotherapy, antimicrobials, coating agents, anesthetics and analgesics, growth factors and cytokines, and natural agents (Elad et al., 2020). Although the scope and depth of research and clinical practice for the management of CTOM has strategically escalated over the past decades, there is no fully effective method to treat or prevent CTOM (Daugėlaitė et al., 2019). In a systematic review by (MASCC/ISOO): Antifungals, sucralfate, doxepin, coating agents (including mucoadhesive hydrogel and polyvinylpyrrolidone) had insufficient evidence to form a guideline, only morphine (topical) was recommended at level Ⅲ of evidence for CTOM in head and neck cancer patients (Saunders et al., 2020). Natural products have shown great promise in the treatment of CTOM caused by cancer therapy (Panahi et al., 2016; Nagi et al., 2018). A meta-analysis indicated that several Chinese patent medicines may be effective in the prevention and treatment for CTOM (Xie et al., 2022).

*Periplaneta americana* is an insect belonging to the genus *Periplaneta* of the family *Blattellidae*. There are no records about *P. americana* in Medical Plant Names Services (http://mpns.kew.org/mpns-portal) or Plants of the World Online (http://www.plantsoftheworldonline.org). The cultivation of *P. americana* for medical purpose should comply with the Good Agricultural Practice for Chinese Crude Drugs (GAP) of the China National Medical Products Administration (NMPA), and *P. americana* for medicine has been included in the Traditional Chinese Medicine Standards of several provinces in China, including Hunan Province (2010), and Sichuan Province (2010).

Kangfuxin liquid (KFXL) is a single formulation of the ethanol extract of *P. americana* dry body as the only ingredient., which has been approved by the NMPA for oral use for blood stasis, stomach pain and bleeding, and gastric and duodenal ulcers. External use: for wounds such as pressure sores, trauma, ulcers, fistulas, burns, scalds and bedsores (Zeng et al., 2019). Besides, KFXL produced by God-doctor panxi pharmaceutical Co., Ltd. has been approved by Health Canada as natural health product to invigorate the blood, dispel blood stasis, and nourish yin. KFXL contains a variety of active ingredients including polyols, fatty acids, peptides, nucleosides, dopamine, amino acids, viscous sugar amino acids, coumarin, uracil, xanthine, inosine, epidermal growth factors. (Ali et al., 2017; Cha et al., 2023; Lu et al., 2022; Zhang et al., 2024). There are no relevant chemical ingredients are included in the TCMSP database, which is the largest noncommercial Traditional Chinese Medicine (TCM) database worldwide (http://ibts.hkbu.edu.hk/LSP/Tcmsp.php). We searched multiple databases, and summarized the active ingredients in Supplementary Table S1.

Chinese expert consensus also recommends TCM for the prevention and treatment of CTOM, and KFXL is one of the recommended TCM (Expert Committee on Safety Management of Anti-Tumor Drugs in Chinese Society of Clinical Oncology, 2021). There are some differences in the management between CTOM and radiotherapy-induced oral mucositis (RTOM), and in this study we will focus on CTOM first. In recent years, a number of clinical trials have shown that KFXL has a good effect in the treatment and prevention of CTOM. However, to date, no meta-analysis has been conducted to fully summarize these research studies to determine whether KFXL is more effective than other basic therapies or other drugs in preventing and treating CTOM. To provide more reliable evidence for the clinical application of KFXL, we conducted this systematic review and meta-analysis of published RCTs to quantitatively assess the clinical effectiveness of KFXL in preventing and treating CTOM.

## 2 Methods

This systematic review and meta-analysis were performed according to the Preferred Reporting Items for Systematic Reviews and Meta-Analyses (PRISMA) guidelines, and registered in the International Prospective Register of Systematic Reviews (PROSPERO) (CRD42024585859).

### 2.1 Search strategies

The following nine electronic databases were searched by two independent researchers for published studies from inception to August 2024: PubMed, Web of Science, Embase, Scopus, The Cochrane Library, China National Knowledge Infrastructure (CNKI), Chinese BioMedical Literature Database (CBM), VIP information and Wanfang Data. The main search terms were as follows: (kangfuxin liquid OR *P. americana*) AND (oral mucositis OR chemotherapy OR cancer).

### 2.2 Inclusion criteria

Inclusion criteria were set according to the PICOS component:

P (population): Patients diagnosed with cancer who are about to receive chemotherapy, or who have developed oral mucositis and received chemotherapy, regardless of age, sex, race, or type of cancer and chemotherapy agent.

I (intervention): Treatment groups received KFXL alone or KFXL in combined with other medicine.

C (comparator): Placebo or any form of medical intervention except KFXL.

O (outcomes): The paper must have reported at least one of the following primary or secondary outcomes. Primary outcomes included: incidence rate, effective rate and cure rate of CTOM or severe CTOM, the grading of CTOM. We defined severe CTOM as Grade Ⅲ and Grade Ⅳ CTOM (either of the two common grading scales: CTCAE, WHO), with treatment to Grade 0 being considered a cure. Secondary outcome was healing time.

S (strategy): RCT design studies in English or Chinese with full text.

### 2.3 Exclusion criteria

Uncontrolled trials, reviews, posters, case reports, secondary research, meeting abstracts, *in vitro* or animal studies, and papers without complete data or with incorrect data.

### 2.4 Paper screening and data extraction

EndNote X7.3 software, which pooled search results from nine databases, was used to screen the literature and remove duplicates. After removing duplicates, two independent researchers pre-screened the titles and abstracts, and then re-screened the full text of all potential papers against the inclusion and exclusion criteria. Two authors independently extracted the relevant and crucial information from the included research using a structured data collection form. The following data were recorded: name of the first author, publication year, study design, number of patients analyzed, details of the clinical intervention, chemotherapeutic agent, scales for CTOM, outcomes, and duration of treatment. Any discrepancies in paper screening and data extraction were resolved by discussion by a third researcher.

### 2.5 Quality assessment

Two independent investigators evaluated the methodological quality of each eligible studies by the Cochrane Risk of Bias Assessment Tool, which considered following seven assessment criteria: random sequence generation, allocation concealment, blinding of participants and personnel, blinding of outcome assessment, incomplete outcome data, selective reporting, and other sources of bias. Any divergent assessment was resolved in open discussion or consultation with the corresponding author. The risk of bias figures were created using RevMan 5.4 software.

### 2.6 Statistical analysis

Statistical analysis was performed for each outcome using RevMan5.4 software. Subgroup analysis was performed based on the type of control treatment. For dichotomous data, such as incidence rate, effective rate, cure rate, were computed as risk ratio (RR). For continuous variables, including healing time, VAS score, were assessed as the mean difference (MD). M-H (Mantel-Haenszel) method was used to analyze the model. Confidence intervals (CIs) were set at 95% to evaluate the effect size. Heterogeneity among studies was evaluated using *I*-squared (*I* ^*2*^) statistics. An *I*-value >50% was indicative of significant homogeneity, and the data were pooled by fixed-effect model; otherwise, random-effects model was chosen. A *p*-value <0.05 was considered significant between the two groups. A funnel plot was performed to assess publication bias when the number of included trials was more than 10, and an Egger test was performed to quantify publication bias using Stata 14.0. *P* < 0.05 was considered statistically significant.

## 3 Results

### 3.1 Search results and study characteristics

**FIGURE 1:**
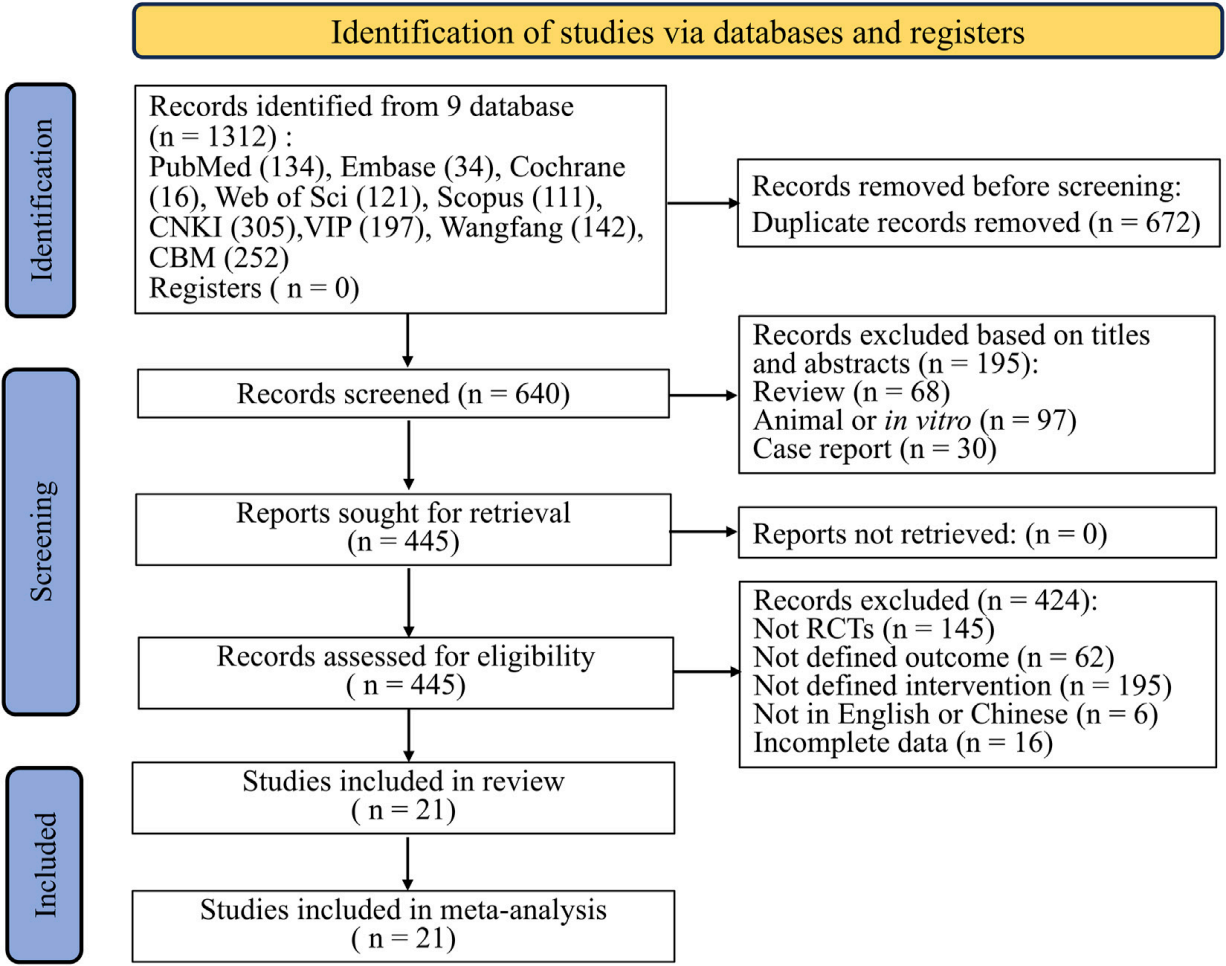
Flowchart of the study selection process.

A total of 21 relevant articles were collected from nine databases following search strategy. After removal of duplicates, initial screening of titles and abstracts, and review of full articles, 21 articles finally met the inclusion criteria and were included in our meta-analysis (Ao and Liu, 2013; Bao, 2013; Cai, 2019; Du, 2019; He, 2021; Hu, 2016; Jiang et al., 2018; Li, et al., 2018; Liu, 2012; Liu, 2018; Liu and Gao, 2016; Qi and Wu, 2017; Qiu et al., 2021; Sun et al., 2024; Tang, 2023; Wei et al., 2022; Wu and Zhang, 2021; Yang et al., 2016; Zeng and Peng, 2019; Zhang et al., 2016; Zhu et al., 2014). The detailed search process is described in Figure 1. The all included trials were conducted in China, with 20 publications were presented in Chinese language and 1 in English. The patients included in our research were diagnosed with various types of cancer, including intestinal cancer, lung cancer, breast cancer, etc. It is worth noting that the patients included in four studies were children with leukemia (Ao and Liu, 2013; Liu, 2012; Qi and Wu, 2017; Zhu et al., 2014). Of the 21 eligible trials, four (Du, 2019; Hu, 2016; Qiu et al., 2021; Zeng and Peng, 2019) and fifteen trials investigated the preventive and therapeutic effects of KFXL on CTOM (Ao and Liu, 2013; Cai, 2019; He, 2021; Jiang et al., 2018; Li et al., 2018; Liu, 2012; Liu, 2018; Liu and Gao, 2016; Qi and Wu, 2017; Sun et al., 2024; Tang, 2023; Wei et al., 2022; Wu and Zhang, 2021; Yang et al., 2016; Zhu et al., 2014), respectively, and two trials (Bao, 2013; Zhang et al., 2016) investigated the therapeutic and preventive effects simultaneously. The usage in instructions of KFXL include oral and topical use. In the included trials, 11 trials were administered by gargling, 9 trials were administered orally after gargling, and 1 trial was administered topically. As recommended in the guidelines (Elad et al., 2020), we define BOC as one or more of the following: patient education, saline rinses, sodium bicarbonate rinses, chlorhexidine rinses, conventional and professional oral hygiene, and conventional nutritional support such as vitamin supplementation. In the included trials, 9 compared the efficacy of KFXL with BOC for CTOM, and 12 compared it with other medical interventions, including tinidazole, interleukin-11, Longzhang oral rinse, Gancao Xiexin decoction, Qianyang Fengsui Dan and montmorillonite powder. The duration of treatment ranged from 5 to 14 days, or one chemotherapy cycle. The detailed characteristics of the eligible trials are shown in Table 1.

**TABLE 1:** Characteristics of included studies.

Study	Cancer type	Purpose	Study design	Simple size (T/C)	Age (T/C)	Grading scales	Chemotherapeutics	Intervention		Usage and dosage	Treatment duration	Outcomes
								Treatment	Control			
Ao and Liu (2013)	Childhood acute lymphoblastic leukemia	Treatment	RCT	26/23	2–11/2–10	NR	Methotrexate	Kangfuxin liquid	BOC (chlorhexidine + vitamin B2)	Swallow after gargle: 20–40 mL	7 days	Cure rate, healing time
Bao (2013)	Breast cancer	Prevention/Treatment	RCT	Prevention: 68/6 Treatment: 10/20	42.2 ± 16.2/41.8 ± 18.6	WHO	Epirubicin + Fluorouracil + Cyclophosphamide	Kangfuxin liquid	BOC (chlorhexidine)	Gargle: 30–40 mL	Chemotherapy cycle	Incidence rate, healing time
Cai (2019)	Oral squamous cell carcinoma	Treatment	RCT	50/50	45.3 ± 2.4/45.9 ± 2.6	WHO	Pingyangmycin	Kangfuxin liquid	BOC (vitamin C + vitamin B2)	Swallow after gargle: 45 mL	7 days	Cure rate, effcacy rate
Du (2019)	Lung cancer	Prevention	RCT	45/45	56.4 ± 5.2/57.4 ± 5.0	WHO	NR	Kangfuxin liquid	BOC	Gargle: 30 mL	Chemotherapy cycle	Incidence rate
He (2021)	Breast cancer	Treatment	RCT	30/30	45.47 ± 3.62/45.18 ± 3.23	NR	Cyclophosphamide	Sijunzi Decoction	Kangfuxin liquid	Swallow after gargle: 30 mL	14 days	Cure rate, effcacy rate
Hu (2016)	Intestinal cancer	Prevention	RCT	61/60	NR	WHO	Oxaliplatin + Fluorouracil + Folinic acid	Kangfuxin liquid	BOC	Swallow after gargle: 30 mL	10 days	Incidence rate
Jiang et al. (2018)	Multiple cancers	Treatment	RCT	50/50	50/53	WHO	NR	Kangfuxin liquid	Interleukin-11	Gargle: 40 mL	7 days	Cure rate, efficacy rate
Li et al. (2018)	Oral squamous cell carcinoma	Treatment	RCT	53/53	NR	WHO	Pingyangmycin	Kangfuxin liquid	BOC (vitamin C + vitamin B2)	Swallow after gargle: 45 mL	7 days	Cure rate, efficacy rate
Liu (2012)	Childhood leukemia	Treatment	RCT	25/20	6.8/6.5	NR	Vincristine + Daunorubicin + L-asparaginase	Kangfuxin liquid	BOC (chlorhexidine)	Swallow after gargle: 15–30 mL	5 days	Cure rate, efficacy rate, healing time
Liu (2018)	Lymphoma	Treatment	RCT	90/90	35.1 ± 10.1	WHO	NR	Kangfuxin liquid	BOC (vitamin B12+saline)	Swallow after gargle: 30–40 mL	7 days	Cure rate, efficacy rate
Liu and Gao (2016)	Gestational trophoblastic neoplasia	Treatment	RCT	25/25	NR	WHO	Methotrexate	Kangfuxin liquid	Tinidazole	Gargle: 40 mL	7 days	Cure rate, efficacy rate, healing time
Qi and Wu (2017)	Childhood acute lymphoblastic leukemia	Treatment	RCT	30/30	5.6 ± 1.4/5.9 ± 1.6	NCI-CTCAE	NR	Kangfuxin liquid	Longzhang oral rinse	Gargle: 12–20 mL	6 days	Cure rate, efficacy rate, healing time
Qiu et al. (2021)	lung cancer	Prevention	RCT	47/47	58.8 ± 8.8/59.0 ± 8.7	WHO	NR	Kangfuxin liquid	BOC	Gargle: 20 mL	Chemotherapy cycle	Incidence rate
Sun et al. (2024)	Breast cancer	Treatment	RCT	51/50	47.19 ± 3.43/47.57 ± 3.16	NR	Paclitaxel	Gancao Xiexin Decoction	Kangfuxin liquid	Gargle: 30–45 mL	7 days	Cure rate, efficacy rate, healing time
Tang L (2023)	Leukemia	Treatment	RCT	51/51	5.8 ± 1.3/6.1 ± 1.5	NR	NR	Kangfuxin liquid	Longzhang oral rinse	Gargle: 12–20 mL	NR	Cure rate, efficacy rate, healing time
Wei et al. (2022)	Multiple cancers	Treatment	RCT	27/23	26–81	WHO	Platinum±5-FU; Paclitaxel	Interleukin-11	Kangfuxin liquid	Gargle: 40 mL	7 days	Cure rate, healing time
Wu and Zhang (2021)	Hematological malignancies	Treatment	RCT	27/27	53.75 ± 15.82	NR	NR	Kangfuxin liquid	BOC (sodium bicarbonate)	Gargle: 60 mL	5 days	Cure rate, efficacy rate, healing time
Yang et al. (2016)	Multiple cancers	Treatment	RCT	48/48	53.51 ± 7.94	NR	Breast cancer: Docetaxel + Epirubicin, Gastric cancer: Docetaxel + Cisplatin, Intestinal cancer: Oxaliplatin + Fluorouracil	Qianyang Fengsui Dan	Kangfuxin liquid	Swallow after gargle: 30 mL	14 days	Healing time
Zeng and Peng (2019)	Leukemia	Prevention	RCT	60/60	40.8 ± 11.2/39.4 ± 10.8	WHO	NR	Kangfuxin liquid	BOC	Gargle: 40 mL	Chemotherapy cycle	Incidence rate
Zhang et al. (2016)	Gastrointestinal tumors	Prevention/Treatment	RCT	Prevention: 32/32 Treatment: 16/25	42.9 ± 4.2/42.8 ± 4.3	WHO	NR	Kangfuxin liquid	BOC	Swallow after gargle: 30 mL	14 days	Incidence rate, healing time
Zhu et al. (2014)	Childhood leukemia	Treatment	RCT	25/25	5.8 ± 1.3/6.1 ± 1.5	WHO	NR	Kangfuxin liquid	Montmorillonite powder	Smear	5 days	Cure rate, efficacy rate, healing time

T: Test group; C: Control group; CTOM: Chemotherapy-induced oral mucositis; KFXL: Kangfuxin liquid; RCT, Randomized controlled trial, BOC: Basic oral care; NR: Not report.

CNKI = China National Knowledge Infrastructure, CBM = Chinese BioMedical Literature Database.

### 3.2 Risk of bias

**FIGURE 2:**
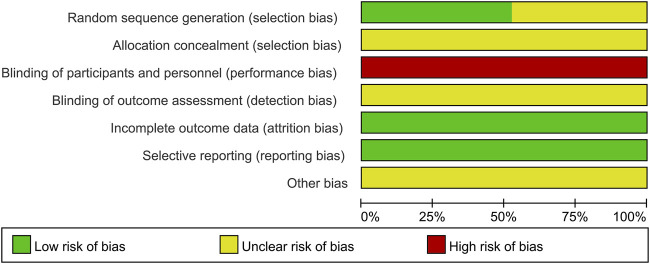
Risk of bias graph.

**FIGURE 3:**
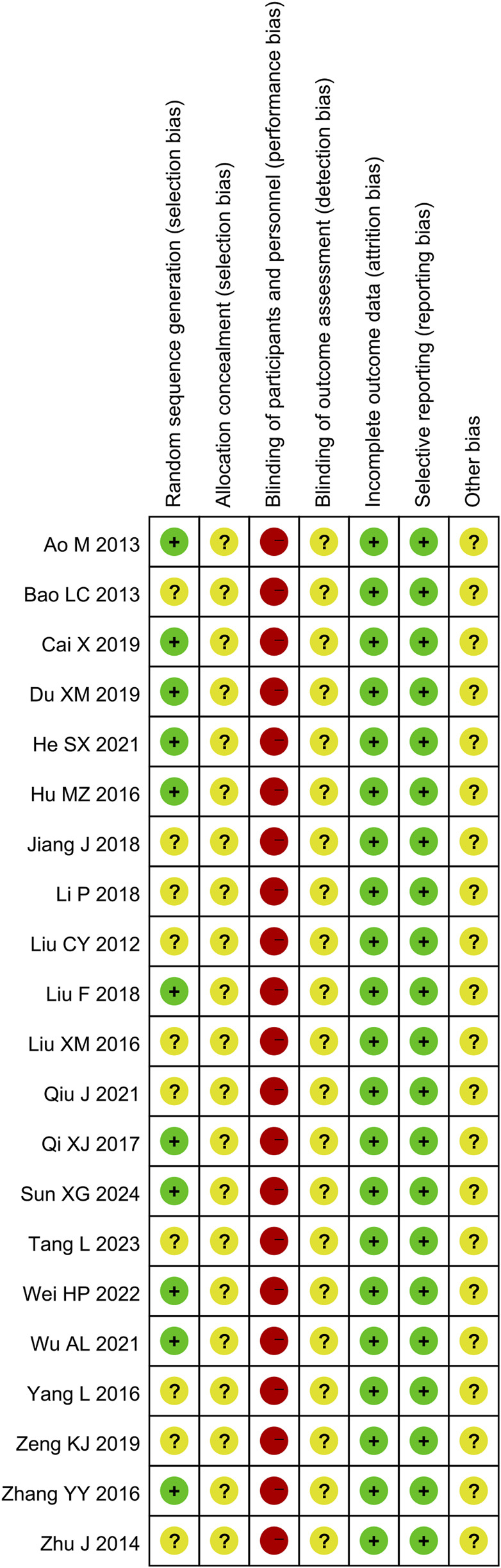
Risk of bias summary.

The methodological quality of the 21 included trials was not high assessed by Cochrane Risk of Bias tool. All the trials reported random allocation, whereas 9 trials did not provide details. There was insufficient information about allocation concealment and outcome assessment blinding, and the risk of both was judged to be unclear. And given the odor, colour and administration methods of KFXL, none of the studies mentioned blinding of patients and participants, we evaluated performance risk is high. All 21 trials had complete outcome data, and all reported pre-defined outcome indicators, so both of attrition and reporting bias were considered as low risk. There was insufficient information of other biases in the included studies, and the risk assessed was unclear. All details of the bias assessment are shown in Figures 2, 3.

### 3.3 Meta-analysis results

We performed subgroup analysis for different treatment regimens in the control groups. These control groups can be divided into two types: BOC group and other medicine group.

#### 3.3.1 Preventive effect of KFXL on CTOM

**FIGURE 4:**
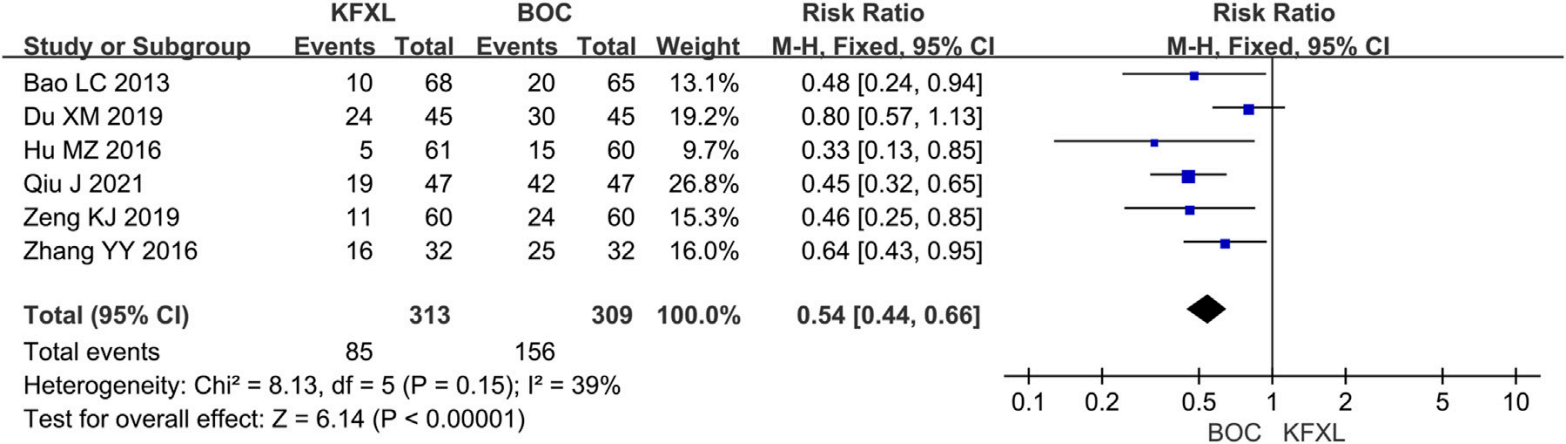
Forest plot of preventive effect of KFXL on CTOM compared with BOC. KFXL: Kangfuxin Liquid; BOC: basic oral care; M-H: Mantel-Haenszel.

Six studies (Bao, 2013; Du, 2019; Hu, 2016; Qiu et al., 2021; Zeng and Peng 2019; Zhang et al., 2016; Liu, 2018) evaluated the effect of KFXL in the prevention of CTOM compared with BOC with low heterogeneity. The analysis results suggested that KFXL significantly reduced the incidence of CTOM. (RR = 0.54, 95% CI: 0.44–0.66, p < 0.00001, *I* ^*2*^ = 39%, fixed-effect model) (Figure 4).

#### 3.3.2 Preventive effect of KFXL on severe CTOM

**FIGURE 5:**
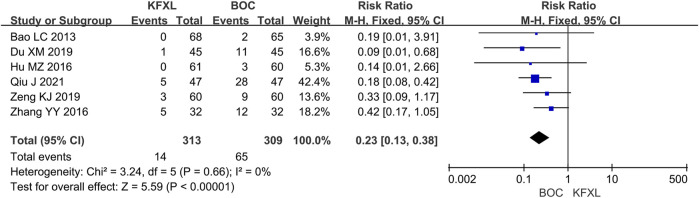
Forest plot of preventive effect of KFXL on severe CTOM compared with BOC. KFXL: Kangfuxin Liquid; BOC: basic oral care; M-H: Mantel-Haenszel.

Six trials (Bao, 2013; Du, 2019; Hu, 2016; Qiu et al., 2021; Zeng and Peng 2019; Zhang et al., 2016) reported the incidence of CTOM between KFXL and BOC without significant low heterogeneity. The results of the meta-analysis indicated that the KFXL group had a significantly lower incidence of severe CTOM. (RR = 0.23, 95% CI: 0.13–0.38, p < 0.00001, *I* ^*2*^ = 0%, fixed-effect model) (Figure 5).

#### 3.3.3 Efficacy rate of KFXL on CTOM

**FIGURE 6:**
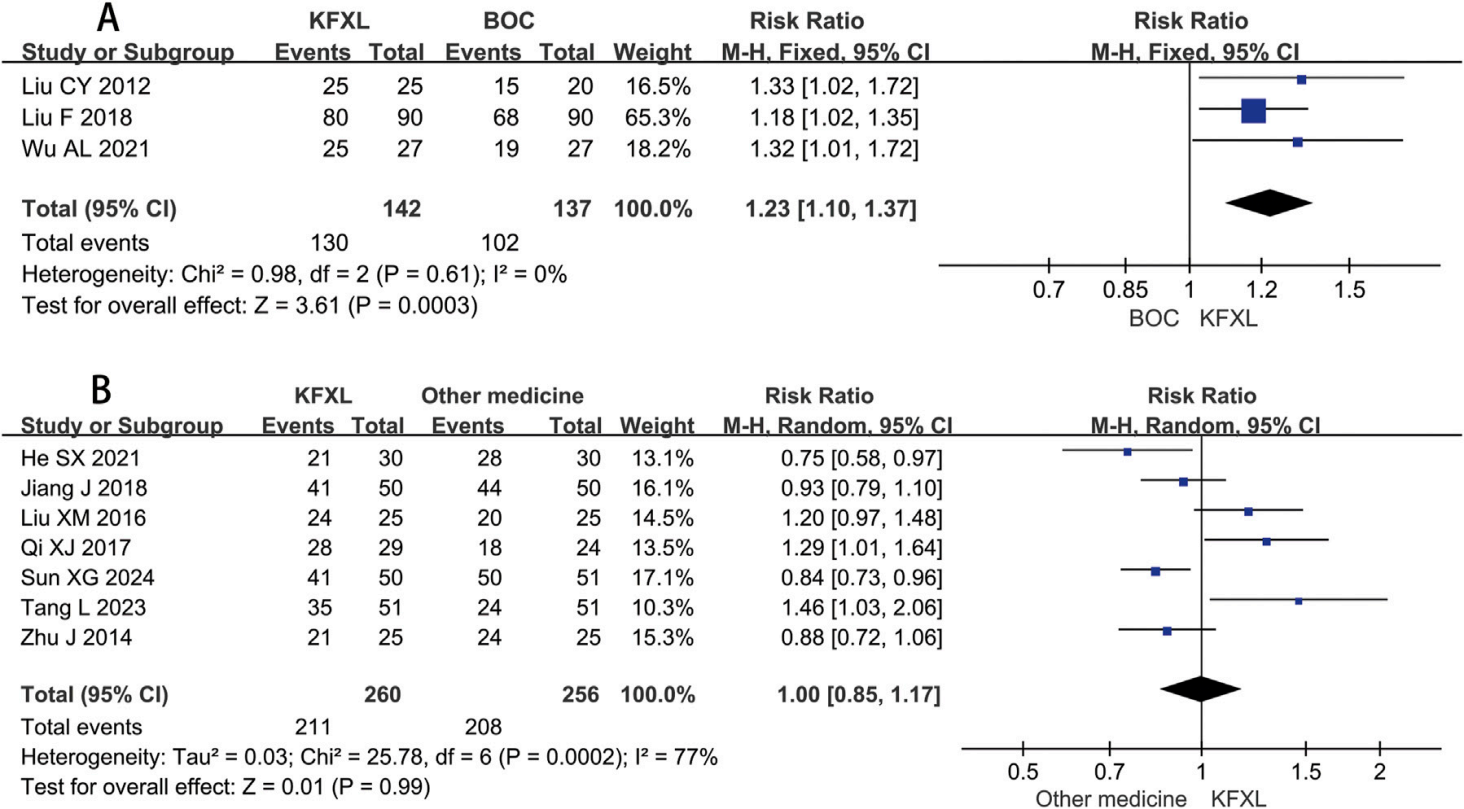
Forest plot of KFXL on efficacy rate on CTOM compared with BOC and other medicines. KFXL: Kangfuxin Liquid; BOC: basic oral care; M-H: Mantel-Haenszel.

Three studies (Liu, 2012; Liu, 2018; Wu and Zhang, 2021) reported on efficacy rate in CTOM comparing KFXL with BOC, pooled data indicated KFXL can significantly increase effective rate compared to BOC (RR = 1.23, 95% CI: 1.10–1.37, p = 0.0003, *I* ^*2*^ = 0%, fixed-effect model) (Figure 6A). Seven studies (He, 2021; Jiang et al., 2018; Liu and Gao, 2016; Qi and Wu, 2017; Sun et al., 2024; Tang, 2023) provided efficacy rate data in CTOM comparing KFXL with other medicines, the analysis results suggested that the KFXL group and the other drugs group had the same therapeutic effect. (RR = 1.00, 95% CI: 0.85–1.17, p = 0.99, *I* ^*2*^ = 77%, random-effect model) (Figure 6B).

#### 3.3.4 Efficacy rate of KFXL on severe CTOM

**FIGURE 7:**
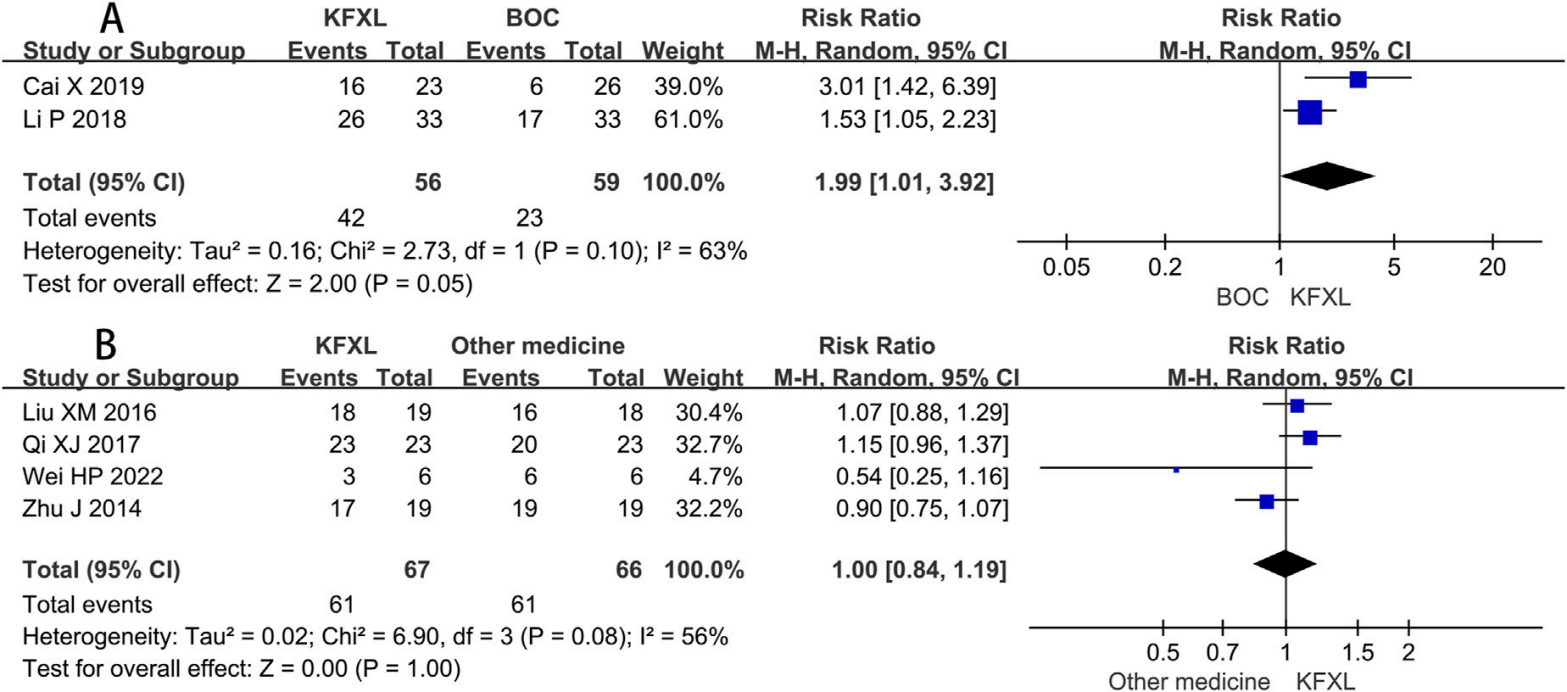
Forest plot of KFXL on efficacy rate on severe CTOM compared with BOC and other medicines. KFXL: Kangfuxin Liquid; BOC: basic oral care; M-H: Mantel-Haenszel.

Only two trials (Cai, 2019; Liu, 2018) compared the efficacy rate between KFXL and BOC in the treatment of severe CTOM, and although this did not reach statistical significance, it implies that the KFXL might be more effective than the BOC. (RR = 1.99, 95% CI: 1.01–3.92, p = 0.05, *I* ^*2*^ = 63%, random-effect model) (Figure 7A). Four trials (Liu and Gao, 2016; Qi and Wu, 2017; Wei et al., 2022; Zhu et al., 2014) compared the effectiveness of KFXL and other medicines in treating severe CTOM, the findings indicated that KFXL and other medicines were equally effective in the treatment of severe CTOM. (RR = 1.00, 95% CI: 0.84–1.19, p = 1.00, *I* ^*2*^ = 56%, random-effect model) (Figure 7B).

#### 3.3.5 Cure rate of KFXL on CTOM

**FIGURE 8:**
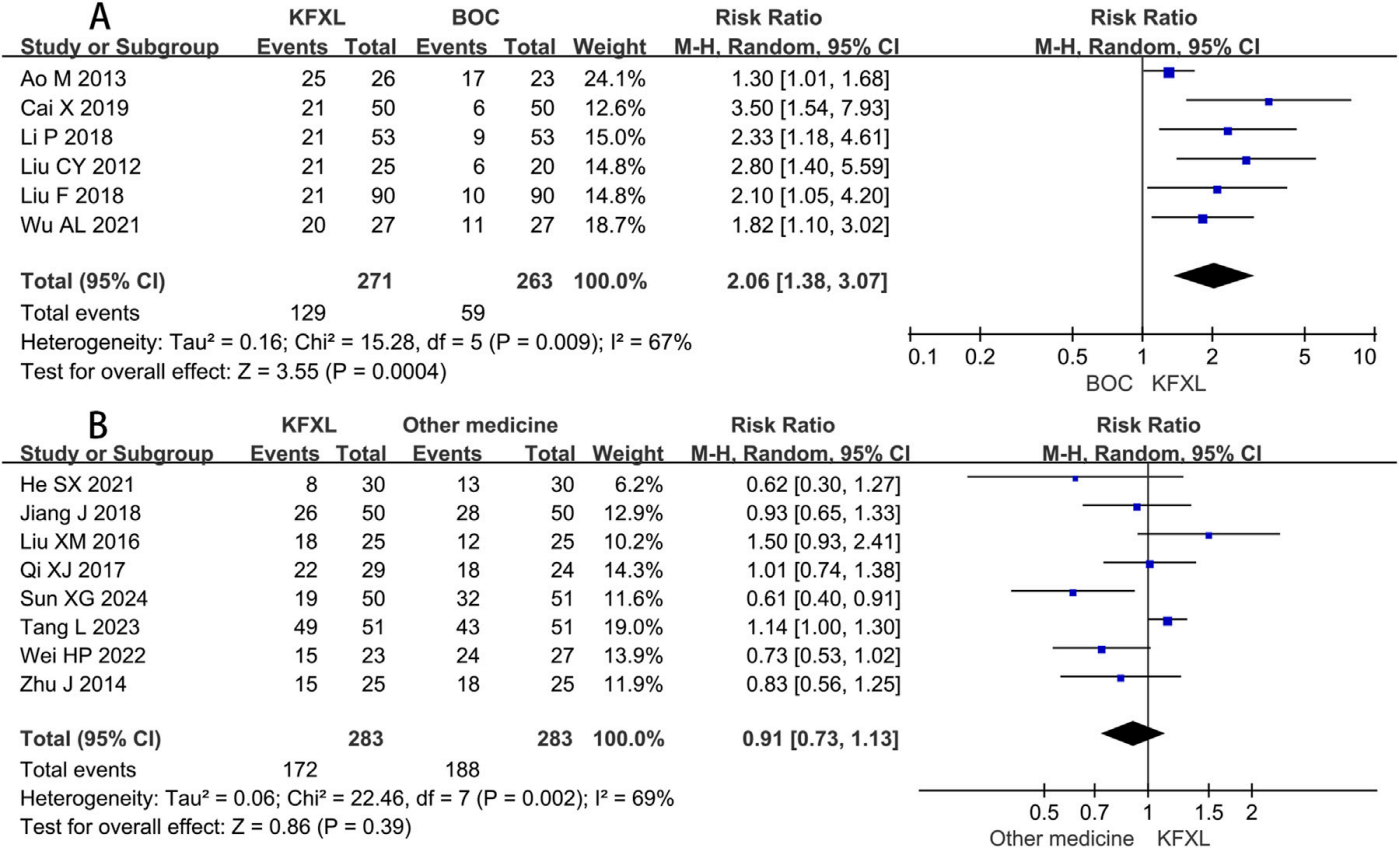
Forest plot of KFXL on cure rate on CTOM compared with BOC and other medicines. KFXL: Kangfuxin Liquid; BOC: basic oral care; M-H: Mantel-Haenszel.

Compared to BOC, pooled data of six studies (Ao and Liu, 2013; Cai, 2019; Li et al., 2018; Liu, 2012; Liu, 2018; Wu and Zhang, 2021) suggested KFXL could raise the number of cured patients with CTOM. (RR = 2.06, 95% CI: 1.38–3.07, p = 0.0004, *I* ^*2*^ = 67%, random-effect model) (Figure 8A). A Meta-analysis including eight trials (He, 2021; Jiang et al., 2018; Liu and Gao, 2016; Qi and Wu, 2017; Sun et al., 2024; Tang, 2023; Wei et al., 2022; Zhu et al., 2014) revealed that there is no significant difference in the efficacy between KFXL and other medicines in cure rate. (RR = 0.91, 95% CI: 0.73–1.13, p = 0.39, *I* ^*2*^ = 69%, random-effect model) (Figure 8B).

#### 3.3.6 Healing time

**FIGURE 9:**
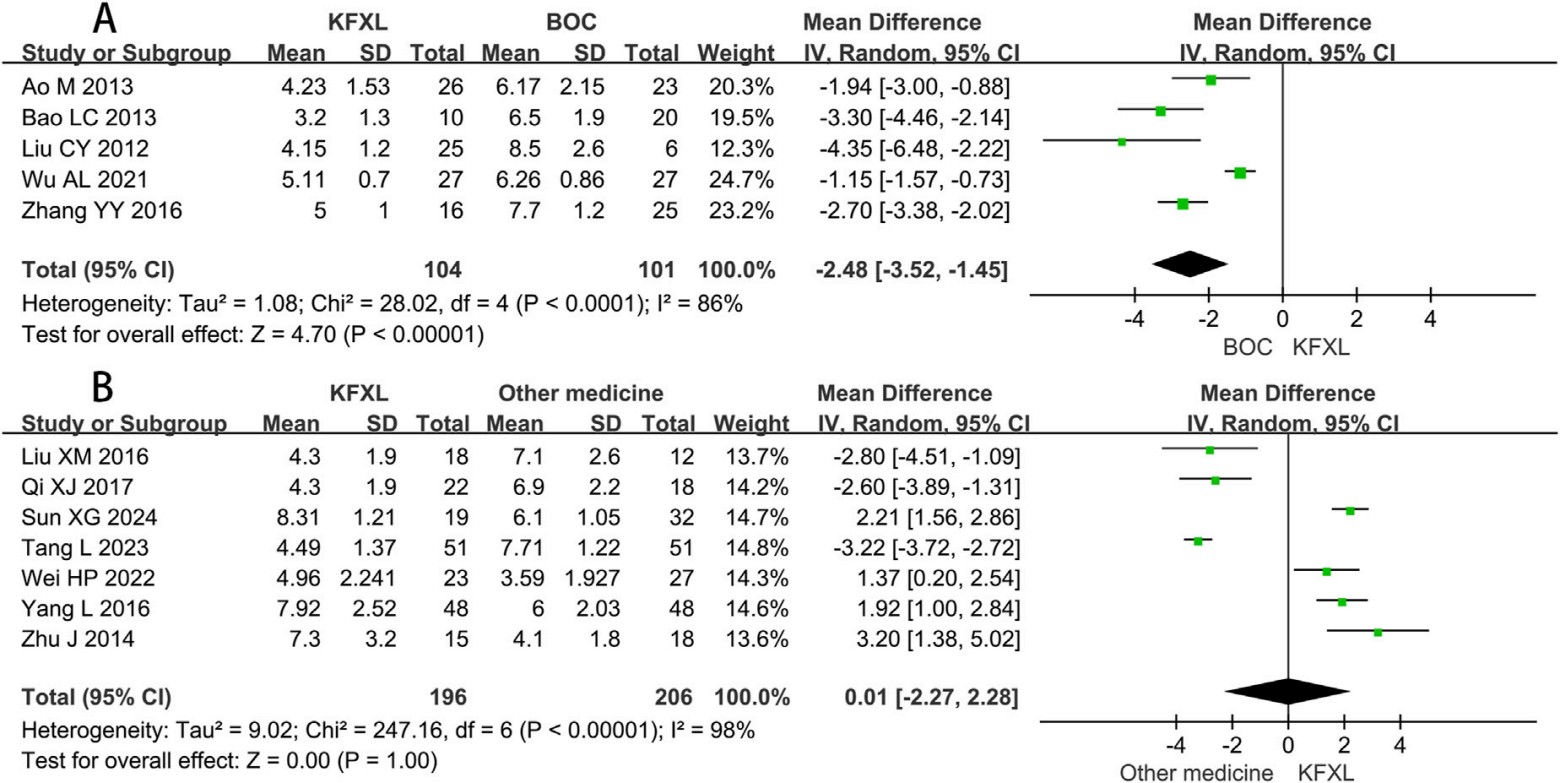
Forest plot of KFXL on healing time on CTOM compared with BOC and other medicines. KFXL: Kangfuxin Liquid; BOC: basic oral care; I-V: Inverse Variance.

Five studies (Ao and Liu, 2013; Bao, 2013; Liu, 2012; Wu and Zhang, 2021; Zhang et al., 2016) provided data on the healing time of CTOM comparing KFXL with BOC, the analysis results suggested that KFXL group was superior in accelerating the healing process compared to the BOC group. (MD = −2.48, 95% CI: −3.52 to −1.45, p < 0.00001, *I* ^*2*^ = 86%, random-effect model) (Figure 9A). Seven studies (Liu and Gao, 2016; Qi and Wu, 2017; Sun et al., 2024; Tang, 2023; Wei et al., 2022; Yang et al., 2016; Zhu et al., 2014) evaluated the healing time of KFXL compared with other drugs, but no significant difference was found between the KFXL and other drug group in healing time of CTOM. (MD = −0.01, 95% CI: −2.27 to 2.28, p = 1.00, *I* ^*2*^ = 98%, random-effect model) (Figure 9B).

### 3.4 Publication bias

Funnel plots were not construct as the number of trials in all comparison groups was less than 10. We performed Egger test to quantify potential publication bias. Significant publication bias was observed in 4 trials (Liu and Gao, 2016; Qi and Wu, 2017; Wei et al., 2022; Zhu et al., 2014) comparing efficacy rate between KFXL and other medicine for severe CTOM (Egger test, p = 0.014), and in 6 trials (Ao and Liu, 2013; Cai, 2019; Li et al., 2018; Liu, 2012; Liu, 2018; Wu and Zhang, 2021) evaluating the cure rate between KFXL and BOC (Egger test, p = 0.001). There was no significant publication bias in other subgroup.

## 4 Discussion

### 4.1 Summary of findings

CTOM is a common adverse effect in cancer patients receiving chemotherapy. Patients with CTOM, especially severe CTOM, may require longer hospital stays, reduced chemotherapy regimens with delayed cancer treatment and poorer prognosis, increased use of opioid to manage oral pain, and use of total parenteral nutrition due to inability to eat or drink (Alsulami and Shaheed, 2022). Our understanding of the pathogenesis of CTOM has also improved. A five-stage biological model was summarized to explain the mechanism of pathogenesis: first, chemotherapy induces cellular damage and the of radical generation, leading to the basal epithelial cell death. This is followed by an increase in inflammatory factors that exaggerate cell death. Upregulation of pro-inflammatory cytokines leads to mucosal ulceration, which accelerates secondary infection. The final stage involves epithelial proliferation and cellular and tissue differentiation (Daugėlaitė et al., 2019). The main goals of CTOM management are to reduce the incidence, intensity and duration of symptoms and to provide symptomatic relief (Lalla et al., 2014).

KFXL is mentioned in the MASCC/ISOO 2020 Clinical Practice Guidelines, but not enough to form a guideline recommendation (Elad et al., 2020). Therefore, we conducted a further study using meta-analysis to investigate the efficacy of KFXL in the prevention and treatment of CTOM in cancer patients. In the included studies, the drugs compared with KFXL included: Sijunzi decoction in 1 study, Interleukin-1 in 2 studies, Tinidazole in 1 study, Longzhang Oral Rinse in 2 studies, Gancao Xiexin Decoction in 1 study, Qianyang Fengsui Dan in 1 study, Montmorillonite Powder in 1 study. Due to the variety of the comparator drugs and the small number of trials of a single drug, we have integrated these trials into other medicine groups for meta-analysis, which inevitably leads to heterogeneity. It should be noted that two trials (Qi and Wu, 2017; Tang, 2023) comparing KFXL and Longzhang Oral Rinse came to opposite conclusions, while the other two trials (Jiang et al., 2018; Wei et al., 2022) comparing KFXL and interleukin-1 in the treatment of CTOM reached consistent results that interleukin-1 was more effective than KFXL.

Overall, the results of the meta-analysis suggest that KFXL may provide more benefit in the treatment and prevention of CTOM and severe CTOM compared to the BOC group, and there was no significant difference between the KFXL group and the other drug group. Therefore, the current study results should be interpreted with caution. In the process of this review study, we found that KFXL also has a significant preventive and therapeutic effects on RTOM in head and neck cancer patients, compared with borax-containing gargle, KFXL significantly reduced inflammatory response, promoted cellular immune function, and improved quality of life (Luo et al., 2016; Yuan et al., 2022). Given the higher incidence of RTOM, this will be become a focus of our future attention.

### 4.2 Possible therapeutic mechanisms of the KFXL

The biological complexity of CTOM involve interactions among disrupted tissue structures, inflammatory infiltrations, and oral microbiome (Chen et al., 2020). KFXL has a range of active ingredients that can play different roles in the prevention and treatment of CTOM. Inflammation is a central feature of destructive ulcerative pathology. The transcription factor NF-κB is a key component activated by Toll-like receptor signaling. It is known to contribute to a variety of inflammatory pathways, including those leading to CTOM (Bowen and Cross, 2023). An RCT showed that KFXL can reduce the NF-κB expression and inflammatory cytokines in the gingival crevicular fluid and enhance the efficacy in patients with orthodontic gingivitis (Li et al., 2023). Periplanetasin-4, a peptide derived from *P. americana*, ameliorated the severe inflammatory responses in the Toxin A-induced mouse enteritis model, rescuing villous disruption and interleukin-6 production (Yoon et al., 2017). Periplanetasin-5 also showed anti-inflammatory activity by inhibiting the generation of NO, COX-2, and the pro-inflammatory cytokines TNF-α and IL-6 induced by lipopolysaccharide (Kim et al., 2020). The oral bacteriome was disrupted during chemotherapy and was independently and strongly associated with the severity of oral mucositis (Hong et al., 2019). The oral microbiota may aggravate cancer treatment-induced mucosal damage by promoting cell apoptosis and pro-inflammatory cytokine production (Stringer and Logan, 2015). Patients treated with chemotherapy have been found to have an overall higher prevalence of *Candida albicans* colonization, inducing mucosal bacterial dysbiosis that promotes invasive infection (Bertolini et al., 2019; Ramla et al., 2016). The KFXL has efficient antifungal activity against vulvovaginal candidiasis in mice in two ways: by inhibiting mycelia growth and development to reduce *C. albicans* colonization, and by promoting the secretion and release of IL-17A and neutrophils to fight *C. albicans* infection (Ma et al., 2022). *Periplaneta americana* brain lysates have potent antibacterial properties (Ali et al., 2017). Mucosal restoration is an important stage in the healing of CTOM, and KFXL had a significant effect on oral mucositis restoration (Feng et al., 2023). A miscellaneous polysaccharide from *P. americana* promotes macrophage M2 polarization, and display anti-inflammatory and pro-repair properties (Xiao et al., 2024). KFXL improved cell proliferation and migration, and repaired cutaneous wounds in an animal model (Liang et al., 2022). Given the symptoms of cancer and the side effects of chemotherapy, we did not systematically evaluate the side effect of the KFXL. Of the trials included in this review, only 2 trials reported adverse effects, with no serious adverse effects, which we have summarised in Supplementary Table S4. While the systematic reviews of KFXL in the treatment of other diseases also reported that it had good safety (Li et al., 2018; Lin et al., 2020).

### 4.3 Limitation

Our study had several limitations. Firstly, the problem of low quality of included studies has always been a matter of concern for us. The most critical limitation was the low methodological quality of most the studies analyzed, due to the slightly fishy odour and brown colour of KFXL, there are difficulties in implementing blinding in practice, none of the included studies reported blinding of participants and personnel, none provide the sufficient details of allocation concealment and outcome assessment blinding. In addition, the 21 included studies came from 20 journals, 7 of which were not sure if they were peer-reviewed, adding to concerns about the quality of the included trials. Second, the heterogeneity among studies was too high, the factors contributing to high heterogeneity included: various cancers and chemotherapy regimens, differences in dosage, usage and duration of KFXL administration, differences in control group and participant characteristics. The inclusion of different drugs in the control group makes interpretation difficult. Third, due to commercial reasons, the specific preparation process of these four manufacturing companies cannot be found in public information. Although the Kangfuxin liquid produced by all the four companies are ethanol extract of the dried body of the *P. americana*, and conforms to the China National Drug Standard, there may be some differences in composition as we are not sure whether the preparation process of the four manufacturing companies is consistent, which complicates the interpretation of the active ingredients in KFXL. Fourth, although an extensive literature search was conducted, all the eligible trials were performed in China, which may introduce regional bias and affect the reliability for other populations. Finally, the reports of safety data, including overall safety profiles, adverse events and drug interactions, are not comprehensive, making it difficult to fully assess the safety of KFXL. Taken together, these limitations affect the reliability of the findings, and results of our study should be interpreted with caution.

## 5 Conclusion

Our study suggests that KFXL may be more beneficial than BOC in the prevention and treatment of CTOM and severe CTOM. However, due to the low quality of the included trial and the diversity of other drug groups, it is difficult to compare Kangfuxin liquid with other therapies. Our research suggests that KFXL may be a promising drug for the prevention and treatment of CTOM. More high-quality, double-blind trials should be conducted in the future, especially comparing KFXL with other guideline-recommended drugs, to further evaluate its preventive and therapeutic effects for CTOM.

## Data Availability

The original contributions presented in the study are included in the article/Supplementary Material, further inquiries can be directed to the corresponding author.
