# Supplementary material for: Efficacy of Kangfuxin liquid for preventing and treating chemotherapy-induced oral mucositis: a systematic review and meta-analysis of randomized controlled trials
Source: Front Pharmacol. 2025 Apr 8;16:1565345. doi: 10.3389/fphar.2025.1565345 (PMC12011798; doi:10.3389/fphar.2025.1565345)
Supplement: Supplementary file 1 [file DataSheet1.docx]

***Supplementary Material***

“Efficacy of Kangfuxin liquid for preventing and treating chemotherapy-induced oral mucositis: a systematic review and meta-analysis of randomized controlled trials”

Wei Sun^1^, Yang Jian^2^, Xiaolin Feng^1^, Minru Zhao^2^, Yuan Liu^1^

^1^ Department of Laboratory Medicine, The General Hospital of Western Theater Command, Chengdu, China

^2^ Department of Clinical Pharmacy, The General Hospital of Western Theater Command, Chengdu, China

Correspondence to: Yuan Liu, Department of Laboratory Medicine, The General Hospital of Western Theater Command, Tianhui Road No 270, Chengdu, Sichuan province, China

Email: liuyuan198231@163.com.

*.*

**Table 1. Compounds of Kangfuxin Liquid or *Periplaneta americana***

| **Study** | **Bioactive compounds** | **Species** | **Preparation** | **Function** |
| --- | --- | --- | --- | --- |
| Ali, S.M. (2016) | Isoquinoline group, chromene derivatives, thiazine groups, imidazoles, pyrrole-containing analogs, sulfonamides, furanones, and flavanones | Periplaneta americana | Insect tissue samples were collected in 500 uL of sterile water in batches of samples obtained from 500 cockroaches. The samples were kept on ice during dissection and treated in an identical manner. An estimate was made by eye/weight to ensure that the mass of each tissue was approximately similar. The samples were subjected to four cycles of freeze-thawing in order to cause cellular disruption and lysis. The thawing period was kept as brief as possible and organ extracts were kept cold (4 °C) before re-freezing. The samples were then homogenized aseptically with a tissue grinder, prior to centrifugation at 10,000g for 30 min at 4 °C. The supernatant (crude extract) was then collected and filtered using a sterilized 0.2-μm pore size filter, and protein concentration was determined using a Bio-Rad Protein Assay antibacterial bioassay testing kit. Finally, lysates were stored at -20 °C until needed for antibacterial bioassay testing. | Antibacterial activity |
| Asad, K. (2024) | Chitin and chitosan | Periplaneta americana | NR | Anti-inflammatory, antipyretic activity |
| Basseri, H. (2019) | Chitosan | Periplaneta americana | Initially, and for deproteinization, 5 g of powder from each cockroach were separately treated with 1M HCl at 100 °C for 24 h and filtered through a 20-mesh sieve and washed with distilled 3 h at room temperature with moderate stirring. Subsequently, the demineralization procedure was carried out by filtration of the treated samples with a 20-mesh sieve and washed with distilled water. The procedure was followed by mixing each sample with 50 ml 1% sodium hypochlorite solution (1%, w/v), kept at room temperature for 3 h with moderate stirring to remove the color of the samples. The obtained chitins were filtered by 20-mesh sieve, washed with distilled water, dried overnight at 60 °C, and then the dry weight was recorded. To remove acetyl group from chitins, the yields were treated with 50% w/w NaOH at 100 °C with moderate stirring for 4 h and washed with distilled water and then ethanol. The obtained chitosan was left to dry at room temperature. The whole process of preparing chitin and chitosan of cockroach batches was performed three times. | Antibacterial/antifungal activity |
| Cha, G.L. (2023) | 20 fatty acids including oleic, linoleic, palmitic, stearic acids, arachidonic acid, linolenic acid, 9(Z)-hexadecenoic acid, myristic acid, et al. | Periplaneta americana | The insect body was placed in an 80 °C oven for 1 h to kill, washed 3 times with distilled water to the surface impurities, dried and ensured that all moisture was removed, the insect body were ground into powder with a grinder, ensuring that the powder particles were as small as. The powder was added to 95% ethanol at a ratio of 1:5 and mixed thoroughly in a reactor, refluxed at 90 for 3 h, and the reaction was stopped and cooled to room temperature before dismantling the device. resulting extract was centrifuged (4000 r/min × 15 min), and the supernatant was collected. The above method was for extraction 3 times, and finally the extract was combined. The extract was concentrated into a paste, then washed with deionized water, and centrifuged to remove the water-soluble part to give purified oil. | Antioxidant activity |
| Kim, I.W. (2020) | periplanetasin-5 | Periplaneta americana | Periplanetasin-5 was synthesized using the solid-phase peptide synthesis method | Anti-Inflammatory |
| Lv, N. (2018) | Uracil, hypoxanthine, xanthine, inosine, protocatechuic acid, Cyclo (Gly-Tyr) | kangfuxin Liquid | 0.22 μm micro-porous filter membrane filtration | HPLC fingerprint |
| Nguyen, T. (2020) | dopamine, coumarin, dipeptide, vitamin, organic acid, amino acid and its metabolites | Periplaneta americana | The periplaneta americana bodies were grinded into powder and mixed with 2 times volume (w/v) of petroleum ether at room temperature. After 24 h of agitating, the mixture was centrifuged at 1500 × g for 7 min and the precipitate was then mixed with 3 times volume of deionized water. After using Ultrasonic cell crusher for 30 min, samples were centrifuged at 13,000×g for 30 min at 4 °C. The supernatant was collected and immediately concentrated by a vacuum freeze drier. The remains named by periplaneta americana extract was filtrated with 0.22 μm filters and then immediately frozen to -80 °C until experiments. | Antipyretic, anti-inflammatory and analgesic |
| Rao, J.Y. (2023) | 64 compounds involve many different classification of compounds, including: amino acids (Acetylcholine, L-Methionine, 3-Methyl-L-tyrosine, L-Methionine), amino acid metabolites (Kynurenic acid, erythro-3-Hydroxy-Ls-aspartate, 3-Methyl-L-tyrosine), neurotransmitters (D-synephrine, Phenylethylamine, 5-Aminopentanoic acid), peptides (Butyryl-L-carnitine, erythro-3-Hydroxy-Ls-aspartate, O-Ureido-L-serine, (S)-2-Propylpiperidine), sugars (L-Rhamnofuranose, Sorbitol), nucleotides ((R)-5,6-Dihydrothymine, 5-Hydroxymethyluracil), organic acids (3-Hydroxybenzoic acid), etc. | Periplaneta Americana | After drying periplaneta americana was powdered. Five kilogram of the powder was mixed with 10 times the amount (0.1 kg/L) of petroleum ether for 24 h to remove lipid components. After recovering the solvent, the defatted powder was added with 75% ethanoland soaked for 48 h in a percolation tank. Then, the powder was extracted in the percolation tank with a flow rate of 3 mL/min, and in total 90 L of 75% ethanol were consumed. Finally, the extract was filtered by brinell funnel, concentrated under reduced pressure, and freeze-dried to get percolation extract, then stored in refrigerator at -80 °C | promote post-stroke neuroregeneration and recovery of neurological functions |
| Song, Q. (2017) | Uracil, hypoxanthine, inosine | Periplaneta americana | The powdered dried periplaneta americana (200 g) was extracted with 90% EtOH (1.2 L) twice at 80 ℃. After solvent evaporation, the ethanol extract was recovered. The extract (20 g) was suspended in water (200 mL) at 80 °C. After filtration through 0.22 𝜇m filter membranes at appropriate. concentrations, it was stored at -20 °C until use. The HPLC-diode array detector was used to study P.americana extraction | wound-healing activity |
| Wu, H.M. (2013) | Lupine alkaloids; uracil; hypoxanthine; guanosine; adenosine | Kangfuxin Liquid | 0. 22 μm micro-porous filter membrane filtration | HPLC fingerprint |
| Wu, J.L. (2023) | Uracil, hypoxanthine, uridine, inosine, guanosine, protocatechuic acid | Periplaneta americana | The dried periplaneta americana dry body (500 g) add in 8 times water for the first decoction, soak 30min, boil, keep simmering and decoct for 30 min, filter through a 200-mesh sieve, cool immediately to room temperature. for the second decoction, add in 6 times water, boil, keep simmering and decoct for 20 min, filter through a 20-mesh sieve, combine two decoctions, cool immediately to room temperature, concentration vacuum at low temperature, freeze-dry the concentrated liquid and pack, | HPLC fingerprint |
| Wu, J.Z. (2023) | Alanine, glycine, tryptophan, threonine, tyrosine, isoleucine, leucine, valine, proline, phenylalanine | Periplaneta americana | Periplaneta americana (100 g) was degreased with petroleum ether, dried in an oven at 60 °C, and crushed to obtain P. americana crude powder. The powder was then screened through a 20 mm mesh and extracted twice with 1000 mL of 70% ethanol, heated at 70 °C for 2 h, and finally collected by freeze-drying after filtration. | Antioxidative activity |
| Xiao, X. (2024) | Polysaccharide | Periplaneta americana | The crushed and dried periplaneta americana powder (20 kg) was defatted with petroleum ether in a ratio of 1:7 (w/v). After degreasing and drying, the powder was extracted twice with 0.1 M NaOH at 55 °C for 3 h each time, and the two extracts were combined. The collected solution was concentrated using a rotary evaporator at 55 °C and then centrifuged at 6000 rpm for 10 min. Absolute ethanol was slowly added to the supernatant while stirring rapidly to achieve a final ethanol concentration of 55 %, and the final solution was stored overnight at 4 °C. Subsequently, the precipitate was collected by centrifugation (6000 rpm, 10 min) and lyophilized to obtain pretreated P. americana crude polysaccharide. Use Sevage reagent (chloroform, 4:1, v/v) was used to remove protein from the periplaneta americana powder solution. The supernatant was collected by centrifugation (8000 rpm, 10 min), and remove the excess Sevage reagent was removed. The polysaccharide solution from Periplaneta americana was decolorized with 30 % H_2_O_2_ and dialyzed for 2 days to remove small molecule impurities in the solution. | Anti-inflammatory and wound-healing activities |
| Xie, Y.C. (2023) | Five indole analogues including tryptophan, tryptamine, 1,2,3,4-tetrahydrogen-β-carboline-3-carboxylic acid, (1S, 3S)-1-methyl-1,2,3,4-tetrahydrogen-β-carboline-3-carboxylic acid, and (1R, 3S)-1-methyl-1,2,3,4-tetrahydrogen-β-carboline-3-carboxylic acid | Periplaneta americana | The periplaneta americana concentrated ethanol-extract liquid (containing 2 g worms/mL) was provided by Good Doctor Company (Chengdu, Sichuan province, China) for HPLC analysis and separation, and was also used for experiments on proliferation-promoting activity and effects on UC in mice. | Cell proliferation activity |
| Yan, Y.M. (2018) | N-containing compounds | Periplaneta americana | Periplaneta americana (30 kg) was powdered and extracted by refluxing with 70% EtOH (3×120 L×2 h) to give a crude extract, which was suspended in water, followed by extraction with EtOAc to afford an EtOAc soluble extract | Wound-healing activity |
| Yang, Y.X. (2015) | Periplanosides A–C | Periplaneta americana | The insects of periplaneta americana (20 kg) were powdered and extracted with 95% EtOH at room temperature for two times, once per week. The extract was concentrated and suspended in H2O, followed by successive partition with petroleum ether, EtOAc, and n-BuOH, respectively. | Stimulate collagen production in human dermal fibroblasts |
| Yoon, I.N. (2017) | Periplanetasin-4 | Periplaneta americana | Periplanetasin-4 was synthesized by AnyGen (Gwang-ju, South Korea) | Inhibits cell toxicities and inflammatory responses |
| Zhang, J.Z. (2023) | N-Acetyldopamine | Periplaneta americana | N-Acetyldopamine oligomers were isolated from the CH2Cl2: CH3OH (2:1) extract 35 of Periplaneta americana, through sequential chromatographic methods including silica gel, Sephadex 36 LH-20, preparative HPLC, and chiral-phase separation. | Anti-inflammatory and vasorelaxant effects |
| Zhang, Y. (2024) | Inosine | Kangfuxin Liquid | Take 200ul sample, add 600ul methanol, swirl for 60 s, centrifuge for 15 min, vacuum drain the supernatant, add 200ul 50 % methanol water to dissolve, and then filter through 0.22um organic filter membrane, ready for machine testing | Anti-inflammatory, antipyretic activity |
| Zhu, J.J. (2018) | Seven compounds including: cyclo-(L-Val-L-Pro), 2-(40-methyl-30-pentene)-6-hydroxymethyl-10-methyl-12-hydroxyl-(2,6,10)-triendodecanic acid, arbutin, 4-Benzyloxy-3-methoxybenzoic acid, (E)-3-Hexenyl-β-D-glucopyranoside, 7-Hydroxycotadeca-noic acid, (S)-2,3-Dihydroxypropyl hexadecanoic acid ester | Periplaneta americana | The powdered dried Periplaneta americana (500 g) was extracted with 90% ethanol (3 L) twice at 80 ℃ | Wound-healing activity |

**Reference:**

1. Ali, S. M., Siddiqui, R., Ong, S. K., Shah, M. R., Anwar, A., Heard, P. J., et al. (2017). Identification and characterization of antibacterial compound(s) of cockroaches (*Periplaneta americana*). *Appl Microbiol Biotechnol*. 101 (1), 253–286.

2. Asad, K., Shams, S., Ibáñez-Arancibia, E., De Los Ríos-Escalante, PR., Badshah, F., Ahmad, F., et al. (2024). Anti-Inflammatory, Antipyretic, and Analgesic Potential of Chitin and Chitosan Derived from Cockroaches (Periplaneta americana) and Termites. *J Funct Biomater*. 21;15(3):80.

3. Basseri, H., Bakhtiyari, R., Hashemi, S. J., Baniardelani, M., Shahraki, H., Hosainpour, Laila. (2019). Antibacterial/Antifungal Activity of Extracted Chitosan from American Cockroach (Dictyoptera: Blattidae) and German Cockroach (Blattodea: Blattellidae). *J Med Entomol*.;56(5):1208-1214.

4. Cha, L.G., Gu, Q.H., Wu, M.Y., Hu, L. (2023). Determination of oil composition and antioxidant activity of Periplaneta American. *J Yunnan Minzu Univ (Natural Sciences Edition).*

5. Kim, I. W., Lee, J. H., Seo, M., Lee, H. J., Baek, M., Kim, M. A., et al. (2020). Anti-Inflammatory Activity of Antimicrobial Peptide Periplanetasin-5 Derived from the Cockroach *Periplaneta americana*. *J Microbiol Biotechnol*. 30 (9), 1282–1289.

6. Lv, N., Wang, J.H., Chen, L.H., Shen, L.G., Li, G.Z., Si, J.Y., et al. (2017). Establishment of HPLC fingerprints of Kangfuxin Liquid and determination of six constituents. *Chin Tradit patent med*. 40 (3): 613-617.

7. Nguyen, T., Chen, X., Chai, J., Li, R., Han, X.Y., Chen, X.X., et al. (2020). Antipyretic, anti-inflammatory and analgesic activities of Periplaneta americana extract and underlying mechanisms. *Biomed Pharmacother.* 123: 109753.

8. Rao, J.Y., Li, H.P., Zhang, H.N., Xiang, X.X., Ding, X.Y., Li, L., et al. (2023). Periplaneta Americana (L.) extract activates the ERK/CREB/BDNF pathway to promote post-stroke neuroregeneration and recovery of neurological functions in rats. *J Ethnopharmacol.* 1:321:117400.

9. Song, Q., Xie, Y., Gou, Q.H., Guo, X.Q., Yao Q, Gou X.J., et al. (2017). JAK/STAT3 and Smad3 activities are required for the wound healing properties of Periplaneta americana extracts. *Int J Mol Med.* Aug;40(2):465-473.

10. Wu, H.M., Xu, F., Liu, L.M., Wang, X.P., Yang, M. (2013). Investigation of UPLC Fingerprint for Kangfuxin Liquid. *Chin J Exper Tradit Med Formul.* 19 (8): 59-62.

11. Wu, J.L., Gao, H., Zeng, Y.Z., Wu, Q.L, Chen, B.T., Li, J.S., et al. (2023). Simultaneous Determination of Six Constituents in Standard Decotion of Periplaneta americana by HPLC. *Chin J Ethnomed Ethnopharm.*32(1): 32-37

12. Wu, J.Z., Zhang, Z., Wu, Q.M, Zhang, L.L., Chen, Z.P., Zhao, H.R., et al. (2023). Antioxidative effect of Periplaneta americana extract on dextran sulfate sodium-induced ulcerative colitis through activation of the Nrf2 signal. *Pharm Biol*. Dec;61(1):949-962.

13. Xiao, X., Zhu, A., He, G., Tokula, S., Yang, Y. T., Qing, Y., et al. (2024). A polysaccharide from *Periplaneta americana* promotes macrophage M2 polarization, exhibiting anti-inflammatory and wound-healing activities. Int J Biol Macromol. 281(Pt 1) 135836.

14. Xie, Y.C., Liang, S.W., Zhang, Y.F., Wu, T.Q., Shen, Y.M., Yao, S., et al. (2023). Discovery of indole analogues from Periplaneta americana extract and their activities on cell proliferation and recovery of ulcerative colitis in mice. Front Pharmacol. 20: 14: 1282545.

15. Yan, Y.M., Xian, B., Zhu, H.J., Qi, J.J., Hou, B., Geng, F.N., et al. (2018). N-containing compounds from Periplaneta americana and their activities against wound healing. J Asian Nat Prod Res. 21(2):93-102.

16. Yang, Y.X., Luo, Q., Hou, B., Yan, Y.M., Wang, Y.H., Tang, J.J., et al. (2015). Periplanosides A-C: new insect-derived dihydroisocoumarin glucosides from Periplaneta americana stimulating collagen production inhuman dermal fibroblasts. J Asian Nat Prod Res. 17(10):988-95
17. Yoon, I. N., Lu, L. F., Hong, J., Zhang, P., Kim, D. H., Kang, J. K., et al. (2017). The American cockroach peptide periplanetasin-4 inhibits *Clostridium* difficile toxin A-induced cell toxicities and inflammatory responses in the mouse gut. *J Pept Sci*. 23 (11), 833–839.

18. Zhang, J.Z., Dong, F.F., Wang, Y.J., Wang, C., Zhang, C.M., Ke. X., et al. (2023). N-acetyldopamine oligomers from Periplaneta americana with anti-inflammatory and vasorelaxant effects and their spatial distribution visualized by mass spectrometry imaging. *J Ethnopharmacol.* 10; 318(Pt B): 116989.

19. Zhang Y, Jia DQ, Wu YP, Xu YQ. (2024). Antipyretic and anti-inflammatory effects of inosine, an active component of Kangfuxin. *Immunobiology*. 229(3):152812.

20. Zhu, J.J., Yao, S., Guo, X., Yue, B.S., Ma, X.Y., Li, J. (2018). Bioactivity-Guided Screening of Wound-Healing Active Constituents from American Cockroach (Periplaneta americana). *Molecules*. 20 ;23(1):101.


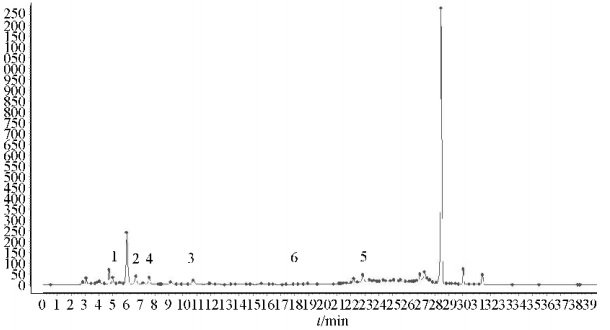


1: Uracil; 2: Hypoxanthine; 3: Xanthine, 4: Inosine; 5: protocatechuic acid; 6: Cyclo (Gly-Tyr)

**Figure 1. HPLC fingerprints of Kangfuxin Liquid of 6 constituents (Lv, N. 2017)**

**
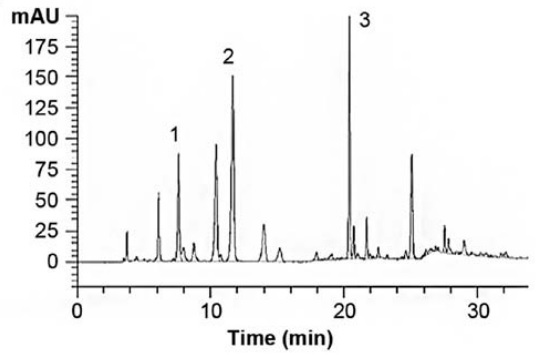
**

1: uracil; 2. Hypoxanthine; 3. Inosine

**Figure 2. Chromatographic separation of Periplaneta americana extract (Song, Q. 2017)**

**
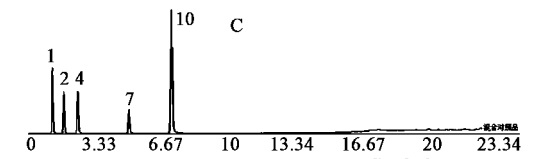
**

1. Lupine alkaloids; 2. Uracil; 4. Hypoxanthine; 7. Guanosine; 10. Adenosine

**Figure 3. HPLC fingerprints of Kangfuxin Liquid of 5 constituents (Wu, H.M. 2013)**

**
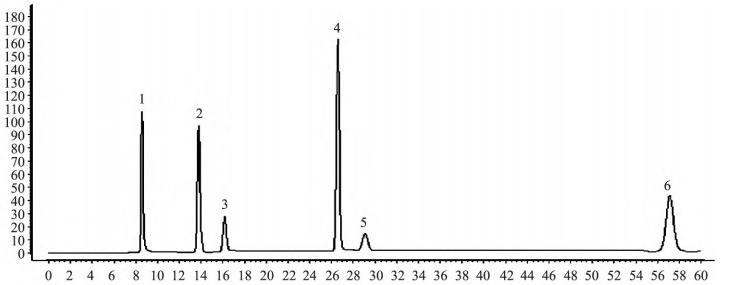
**

1: uracil; 2: Hypoxanthine; 3: Uridine; 4: Inosine; 5: Guanosine; 6: protocatechuic acid

**Figure 4. HPLC of Six Constituents in Standard Decoction of Periplaneta americana (Wu, J.L. 2023)**

**
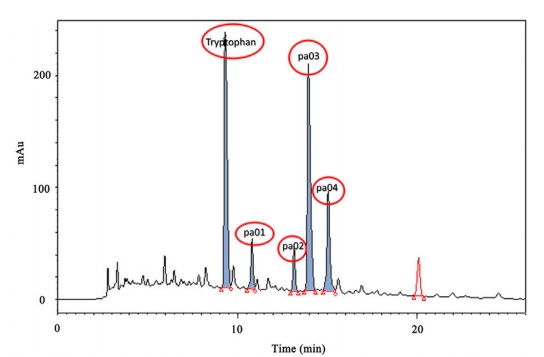
**

1: tryptophan; Pa01: tryptamine; Pa02: 1,2,3,4-tetrahydrogen-β-carboline-3-carboxylic acid;

Pa03: (1S, 3S)-1-methyl-1,2,3,4-tetrahydrogen-β-carboline-3-carboxylic acid,

Pa04. (1R, 3S)-1-methyl-1,2,3,4-tetrahydrogen-β-carboline-3-carboxylic acid

**Figure 5. HPLC gradient elution image of *Periplaneta americana* extract (Xie, Y.C. 2024)**

**
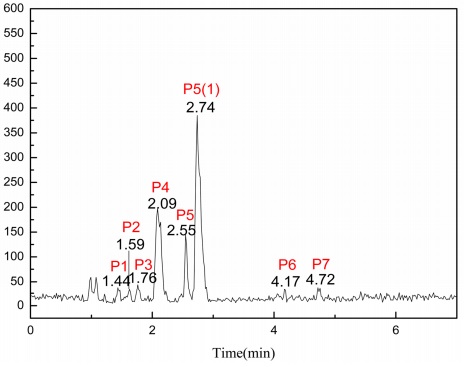
**

P1**:** cyclo-(L-Val-L-Pro); P2: 2-(40-methyl-30-pentene)-6-hydroxymethyl-10-methyl-12-hydroxyl-(2,6,10)-triendodecanic acid; P3: Arbutin; P4: 4-Benzyloxy-3-methoxybenzoic acid; P5: (E)-3-Hexenyl-β-D-glucopyranoside; P6: 7-Hydroxycotadeca-noic acid; P7: (S)-2,3-Dihydroxypropyl hexadecanoic acid ester

**Figure 6. UPLC total ion chromatogram of seven peaks at 254 nm (Zhu, J.J. 2018)**


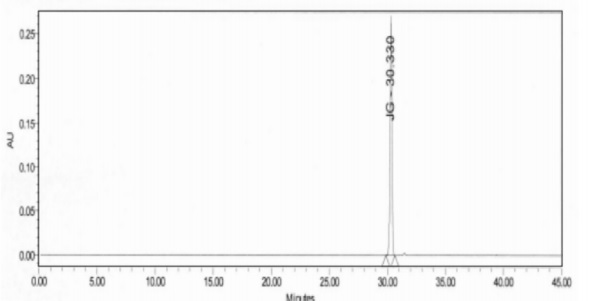


Inosine

**Figure 7. High performance liquid chromatography mass spectrometry peaks of inosine in Kangfuxin Liquid (Zhang, Y. 2024)**

**Table 2. Detailed description of Kangfuxin liquid**

| Component | Ethanol extract of Periplaneta americana dry body |
| --- | --- |
| Description | light brown liquid, a slightly fishy odor and a sweet taste. |
| Indications | Invigorate the blood, dispel blood stasis, and nourish yin.  Oral use: for blood stasis, stomach pain and bleeding, and gastric and duodenal ulcers.  External use: for wounds such as pressure sores, trauma, ulcers, fistulas, burns, scalds and bedsores |
| Usage and Dosage | Oral administration: 10ml each time, 3 times a day, or as directed by a doctor;  External use: apply the drug soaked medical gauze to the affected area, wash the infected wound with this product after debridement, and pack or apply the gauze soaked with this drug. |
| Adverse Reactions | The monitoring data showed that the following adverse reactions were observed:  (1) Digestive system: nausea, vomiting, abdominal distention, abdominal pain, diarrhea, etc.  (2) Application site: redness, swelling, pain, etc.  (3) Skin: rash, pruritus, etc.  (4) Other: dizziness, headache, flushing, dyspnea, allergic reactions, local numbness, anaphylactic shock and other case reports. |
| Contraindications | 1. Contraindicated for those who are allergic to this product and its ingredients.  2. Contraindicated for patients with asthma.  3. Contraindicated for pregnant women. |
| Executive Standard | National Drug Standard WS3-B-3674-2000(Z) |

**Table 3. Information on the Kangfuxin Liquid in the included RCTs**

| No | Study ID | Manufacturing company | Quality control reported? (Y/N) | Chemical analysis reported? (Y/N) | Executive Standard |
| --- | --- | --- | --- | --- | --- |
| 1 | Ao M 2013 | Sichuan Good Doctor Panxi Pharmaceutical Co., Ltd | N | N | WS3-B-3674-2000(Z) |
| 2 | Bao LC 2013 | Inner Mongolia Jingxin Pharmaceutical Co., Ltd | N | N | YBZ00492021 |
| 3 | Cai X 2019 | NR | N | N | NR |
| 4 | Du XM 2019 | NR | N | N | NR |
| 5 | He SX 2021 | Sichuan Good Doctor Panxi Pharmaceutical Co., Ltd | N | N | WS3-B-3674-2000(Z) |
| 6 | Hu MZ 2016 | NR | N | N | NR |
| 7 | Jiang J 2018 | NR | N | N | NR |
| 8 | Li P 2018 | NR | N | N | NR |
| 9 | Liu CY 2012 | Sichuan Good Doctor Panxi Pharmaceutical Co., Ltd | N | N | WS3-B-3674-2000(Z) |
| 10 | Liu F 2018 | Sichuan Good Doctor Panxi Pharmaceutical Co., Ltd | N | N | WS3-B-3674-2000(Z) |
| 11 | Liu XM 2016 | Sichuan Good Doctor Panxi Pharmaceutical Co., Ltd | N | N | WS3-B-3674-2000(Z) |
| 12 | Qi XJ 2017 | Sichuan Good Doctor Panxi Pharmaceutical Co., Ltd | N | N | WS3-B-3674-2000(Z) |
| 13 | Qiu J 2021 | NR | N | N | NR |
| 14 | Sun XG 2024 | Hunan Kelun Pharmaceutical Co., Ltd | N | N | WS3-B-3674-2000(Z) |
| 15 | Tang L 2023 | Inner Mongolia Jingxin Pharmaceutical Co., Ltd | N | N | YBZ00492021 |
| 16 | Wei HP 2022 | NR | N | N | NR |
| 17 | Wu AL 2021 | Hunan Kelun Pharmaceutical Co., Ltd | N | N | WS3-B-3674-2000(Z) |
| 18 | Yang L 2016 | Sichuan Good Doctor Panxi Pharmaceutical Co., Ltd | N | N | WS3-B-3674-2000(Z) |
| 19 | Zeng KJ 2019 | NR | N | N | NR |
| 20 | Zhang YY 2016 | Kunming Sino Pharmaceutical Co., Ltd | N | N | WS3-B-3674-2000(Z) |
| 21 | Zhu Q 2014 | NR | N | N | NR |

**Table 4. Adverse reaction in included studies**

| Study | Intervention | | Adverse event | |
| --- | --- | --- | --- | --- |
|  | Control group | Test group | Control group | Test group |
| Hu, S.X. 2021 | Kangfuxin Liquid | Sijunzi Decoction | 3 patients had gastrointestinal reaction, 2 patients had abnormal liver and kidney function, 2 patients had phlebitis | 1 patient had gastrointestinal reaction |
| Liu, X.M. 2016 | Tinidazole | Kangfuxin Liquid | No | A very small number of patients have nausea reactions due to the smell of the Kangfuxin Liquid at the first dose |
